# Mechanically Reinforced, Flexible, Hydrophobic and UV Impermeable Starch-Cellulose Nanofibers (CNF)-Lignin Composites with Good Barrier and Thermal Properties

**DOI:** 10.3390/polym13244346

**Published:** 2021-12-12

**Authors:** Yadong Zhao, Christofer Troedsson, Jean-Marie Bouquet, Eric M. Thompson, Bin Zheng, Miao Wang

**Affiliations:** 1 School of Food and Pharmacy, Zhejiang Ocean University, Zhoushan 316022, China; yadong@kth.se (Y.Z.); 6369958@163.com (B.Z.); 2 Ocean TuniCell AS, P.O. Box 12, 5868 Blomsterdalen, Norway; christofer.troedsson@oceantunicell.com (C.T.); jean-marie.bouquet@oceantunicell.com (J.-M.B.); eric.thompson@oceantunicell.com (E.M.T.); 3 School of Engineering Sciences in Chemistry, Biotechnology and Health, Royal Institute of Technology, 10044 Stockholm, Sweden; 4 Department of Biological Sciences, University of Bergen, 5006 Bergen, Norway; 5 School of Chemistry and Chemical Engineering, South China University of Technology, Guangzhou 510640, China

**Keywords:** cellulose nanofibers, starch, lignin, composite film, high performance

## Abstract

Bio-based composite films have been widely studied as potential substitutes for conventional plastics in food packaging. The aim of this study was to develop multifunctional composite films by introducing cellulose nanofibers (CNF) and lignin into starch-based films. Instead of costly and complicated chemical modification or covalent coupling, this study optimized the performance of the composite films by simply tuning the formulation. We found that starch films were mechanically reinforced by CNF, with lignin dispersing as nanoparticles embedded in the matrix. The newly built-up hydrogen bonding between these three components improves the integration of the films, while the introduction of CNF and lignin improved the thermal stability of the starch-based films. Lignin, as a functional additive, improved hydrophobicity and blocked UV transmission. The inherent barrier property of CNF and the dense starch matrix provided the composite films with good gas barrier properties. The prepared flexible films were optically transparent, and exhibited UV blocking ability, good oxygen-barrier properties, high hydrophobicity, appreciable mechanical strength and good thermal stability. These characteristics indicate potential utilization as a green alternative to synthetic plastics especially for food packaging applications.

## 1. Introduction

Synthetic polymers, especially petroleum-based plastics as food packaging materials, play critical roles in our daily life and the development of society. However, due to inherent non-degradability in nature, plastics cause serious land and marine pollution, threatening the health of humans and other animals [1]. Nevertheless, finding suitable replacements for synthetic plastics is still a challenge [2]. Recently, many bio-based materials have been chosen as potential candidates to replace synthetic plastics due to their abundance, low cost, renewability and biodegradability [3]. Starch, cellulose and lignin are three such biopolymers that are currently under intensive investigation.

Starch is composed of glucose units with α-1,4 linkages. It is a low cost material and abundantly available in large quantities from several renewable plant sources [4]. Starch often appears in a mixture of two glucosidic macromolecules with a difference in structure and properties: largely linear amylose of molecular weight between 1000 and 1,000,000, consisting of α-(1,4)-linked D-glucose, and amylopectin, having the same backbone as amylose but with a myriad of α-(1,6)-linked branch points [5]. Due to its excellent solubility and film-forming ability, starch is a promising material for production of biodegradable plastics with high potential in food and non-food applications and drug delivery [6,7]. However, pure starch lacks strength, water resistance, processability and thermal stability. To overcome some of these drawbacks, starch is often blended with other polymers. Since the 1970s, starch has been incorporated into polyethylene in order to increase biodegradability [8]. Also, blends of starch with poly (vinyl alcohol) [9], polyethylene [10], polylactic acid [11], polycaprolactone [12] and gelatin [13] have been utilized to form biofilms with improved gas barrier and mechanical properties.

Cellulose, composed of β-1,4-D-glucopyroanose units linearly arranged in elementary fibrils is the most abundant biopolymer in nature. The most common source of cellulose is plants, but among animals, tunicates are the only group able to produce cellulose [14]. This has arisen from lateral gene transfer of a prokaryotic cellulose synthase gene at the base of the tunicate lineage. Recently, some tunicate species, such as *Ciona intestinalis*, have been successfully farmed along the Norwegian western coast with high production capability, indicating the practical utilization of tunicate cellulose at industrial scales (www.oceantunicell.com) [15]. Compared to woody cellulose, tunicate cellulose has a high degree of polymerization (up to 4200), high purity (99%), high crystallinity index (89%), high thermal stability (degradation onset temperature of 207–269 °C), large specific surface area (133 m^2^/g), large aspect ratio (diameter of 10–20 nm and length of several micrometers) and strong mechanical properties (elastic modulus of ~150 GPa) [14]. In addition, further processing of tunicate cellulose to cellulose nanofibers (CNF) through mechanical, enzymatic or chemical approaches not only improves its processability but also renders many specific profiles, such as large aspect ratio, high crystallinity index and high specific surface area [16]. Tunicate CNF as a reinforcing phase has been developed to prepare high-performance composites by blending with either natural or synthetic polymers, such as konja glucomannan [16], silk fibroin [17], polypyrrole [18] and epoxy resin [19].

Another bio-based polymer, lignin, is a complex group of phenolic polymers, available in large quantities as a byproduct of the pulp and paper industry. Recently, research interest in lignin has arisen due to increased focus on biorefineries [20]. The amphilic profile of lignin makes it particularly suitable for biomaterials applications. Lignin is compatible with both organic and inorganic polymers, such as PEO and PET [21], polypropylene [22], polyethylene [22], polyurethane [23], chitin [24] and gelatin [25]. The incorporation of lignin often enhances the mechanical, thermal, and gas barrier properties of polymeric matrices.

Cellulose has been used to reinforce starch-based composites due to its high crystallinity and high aspect ratio, with the starch cementing the cellulose network as a plasticizer [5,26,27,28,29,30]. Furthermore, water vapor barrier properties [30] and thermal stabilities [5] also increase due to the increased crystallinity of the starch-based films. Lignin has been blended with starch to prepare compatible composites with lignin acting either as a filler or as an extender of the starch matrix [31]. This compatibility is favored by relative humidity of media, high amylopectin/amylose ratios and by the presence of the low molecular weight lignin components. For lignosulfonate, the presence of polar sulfonic groups is likely to form hydrogen bonds with amylose and amylopectin hydroxyls, which allows lignosulfonates to form an intimate blend with starch. For non-sulfonated kraft lignin, it leads to composite materials filled with high molecular weight lignin particles and plasticized by the low molecular weight phenolic fraction. The hydrophobic character of kraft lignin imparts improved water resistance of starch films that is more pronounced in the case of cast films [31]. Additionally, mechanical and thermal properties of the biofilms have been reported to be enhanced by lignin addition due to the plasticizing effect of the low molecular weight lignin [32,33]. 

Since either cellulose or lignin can improve the performance of starch films, a cellulose-lignin-starch multifunctional composite could yield improved properties. On the one hand, cellulose could provide good reinforcement, gas barrier properties and thermal stability to starch films. On the other hand, lignin can improve the hydrophobicity and UV blocking ability of starch films. However, none of them could melt, meaning that they are not able to be processed like thermoplastic polymers, making the process different from those using synthetic polymers. In order to overcome this drawback, a pioneering work by Wu et al. (2009) [34], dissolved cellulose, starch and lignin in an ionic liquid, 1-allyl-3-methylimidaxolium chloride (AmimCl), followed by coagulation with water to form composite films. It has been found that cellulose and lignin can improve the mechanical properties of films significantly, while the starch will contribute to the flexibility. The composite films are amorphous with good transparency and gas barrier properties. However, the ionic liquid as well as its recycling procedure are relatively expensive, limiting the large-scale production of such films. In addition, after dissolution, the crystal structure of the cellulose was compromised and the mechanical properties therefore significantly deteriorated.

In this study, we prepared CNF–starch–lignin composite films by a simple blending-casting-evaporation method in an aqueous system, in which the mechanical reinforcement of CNFs was preserved. By simply tuning the formulation, the composite films have acceptable transparency, improved hydrophobicity, strong mechanical strength, good thermal stability and are barriers to UV light and oxygen permeability. They are renewable, biodegradable, edible, and can be used as an alternative to plastic materials in food packaging applications. Moreover, no chemical reaction was involved in the preparation, which makes the process green, simple, cost efficient and scalable.

## 2. Materials and Methods

### 2.1. Materials

Tunicate cellulose was prepared from *Ciona intestinalis* and characterized in our laboratory (Ocean TuniCell AS, Bergen, Norway) [14]. Commercial starch (water soluble, 80% amylopectin and 20% amylose, Sigma S-9765) with a molecular weight of 342.30 was used directly without any further chemical treatment. Softwood kraft lignin was produced at Innventia AB from industrial black liquor following the LignoBoost^®^ process. All reagents were of analytical grade, and they were obtained from VWR International AB, Stockholm, Sweden.

### 2.2. Preparation of Cellulose Nanofibers (CNF)

Never-dried tunicate cellulose was firstly subjected to disintegration (Frank-PTI GmbH, Germany) for 10 min (30,000 revolutions). Then the pretreated cellulose was mechanically disintegrated through high pressure homogenization by using a Microfluidizer (M-110EH, Microfluidics Corp., Westwood, MA, USA) at 10 g/L using two large chambers in series (400 and 200 µm, respectively) at 925 bar for the first pass and smaller chambers (200 and 100 µm, respectively) at 1600 bar for five passes. The obtained CNF dispersed in water were diluted to 0.5% in weight for further use.

### 2.3. Fabrication of Neat Films and Composite Films

A blending-casting-evaporation method was applied to prepare the biocomposites. Briefly, the 0.5% CNF suspension prepared above, starch dissolved in water (0.5%) or lignin dissolved in acetone/water (4:1, *v*/*v*) with a concentration of 0.5% were directly cast in Petri dishes and dried at 50 °C overnight to make neat films. The composite films were prepared by mixing the designated amount of CNF, starch or lignin as shown in Table 1. After sonication for 5 min, the mixed suspension was cast in Petri dishes to obtain films. The films were then dried at 50 °C overnight. Different film compositions were labelled by abbreviations as shown in Table 1. In Table 1, C, S and L stood for CNF, starch and lignin respectively, and the number after L indicated the lignin percentage. For example, SCL25 composite film had 25% lignin.

### 2.4. Characterization Methods

#### 2.4.1. Morphological Analysis

Before scanning electron microscopy (SEM) analysis, all samples were coated with gold using a Cressington 208HR high-resolution sputter coater. A Cressington thickness monitor control thickness to 3–5 nm. Sample morphology was then analysed using a Hitachi S-4800 Field Emission Scanning Electron Microscope.

#### 2.4.2. Fourier Transform Infrared Spectroscopy (FTIR) Analysis

Fourier transform infrared spectra were obtained using a Perkin-Elmer Spectrum 2000 FTIR spectrometer (Waltham, MA, USA) equipped with an ATR system, Spectac MKII Golden Gate (Creecstone Ridge, GA, USA). Samples were analysed at wavelengths ranging from 600–4000 cm^−1^. All spectra were obtained from dry samples subjected to 16 scans at a resolution of 4 cm^−1^ and an interval of 1 cm^−1^ at room temperature. Before data collection, background scanning was performed for background correction.

#### 2.4.3. X-ray Diffraction (XRD) Analysis

A PANalytical X’Pert PRO Materials Research Diffractometer equipped with an X’Celerator detector was used to determine the crystallinity index (CI) of the samples. The analysis was performed using monochromatic C_u_K_α_ radiation at 30 mA and 40 kV. CI, defined to evaluate the crystallinity of the different samples, was calculated using the following equation:

$$\mathrm{CI}\;\left(\%\right)=\frac{I_{200}-I_{\mathrm{am}}}{I_{200}}\times 100$$

where I_200_ is the intensity of the 200 lattice plane at 2θ = 22.8°, and I_am_ is the intensity from the amorphous phase at approximately 2θ = 18° [35].

#### 2.4.4. Contact Angle Determination

The contact angle (CA) was determined by the pendant drop method with a water drop and an optical contact angle meter SL 100B from Solon Information Technology Co., Ltd. (Shanghai, China) at relative humidity (RH) of 50% and 23 °C. To compare different samples, each contact angle was taken at 45 s, and the average value of at least three measurements was used.

#### 2.4.5. Ultraviolet–Visible (UV–Vis) Transmittance Determination

The percent light transmission (T%) of the films was monitored using a Shimadzu UV-240 (Japan) at 750 nm. Film specimens were cut into rectangles and placed in a spectrophotometer test cell directly, and air was used as the reference. Transmittance (T% = I/I_0_, where I and I_0_ were the intensities of emergent and incident radiation, respectively) was used to define the transparency of a film.

#### 2.4.6. Mechanical Strength Measurement

The tensile strength and Young’s modulus of the films were determined using an Instron 4411 mechanical property tester with a 500-N load cell (Instron Ltd., Norwood, MA, USA). The initial grip distance was 25 mm, and the rate of grip separation was 5 mm/min. Two films of each type and three specimens from each film were tested. The specimens were 5 mm wide and approximately 60 mm long. The thickness of the specimens was measured at three points using a micrometer (NSK, Japan).

#### 2.4.7. Oxygen Permeability

Oxygen permeability was measured on a Mocon OXTRAN 2/20 instrument from Modern Controls Inc., USA, equipped with a coulometric sensor, according to ASTM standard D-3985-05. Samples were sealed between aluminium foil with an open area of 5 cm^2^ and then stored in a conditioning room for 1 week (23 °C and 50% RH) prior to analysis. Each sample thickness was determined using a Mitutoyo digital micrometer by taking the average of 5 discontinuous spots. Permeability measurements were carried out twice for each film at 23 °C and 50% RH at atmospheric pressure, and the mean data were reported in cm^3^·μm/m^2^·day·kPa.

#### 2.4.8. Thermo Gravimetric Analysis (TGA)

Thermo gravimetrical analysis was collected using a Mettler Toledo TGA/SDTA 851e equipped with STARe software for data analysis. The samples were subjected to a heating scan between 30 and 800 °C, with a rate of 10 °C/min under an inert atmosphere of nitrogen at a gas flow of 50 mL/min.

## 3. Results and Discussion

### 3.1. CNF Preparation and Characterization

In nature, tunicate cellulose is originally present in the tunic as a composite with protein, lipids and other non-cellulose polysaccharides. After a unique acid hydrolysis-kraft cooking-bleaching procedure, tunicate cellulose in pulp form could be obtained from tunics with a high glucose content (>99%). After enzymatic treatment, the tunicate cellulose was subjected to high pressure homogenization and was disintegrated into elementary fibrils or microfibrillar aggregates, termed tunicate cellulose nanofibers (CNF).

**Figure 1:**
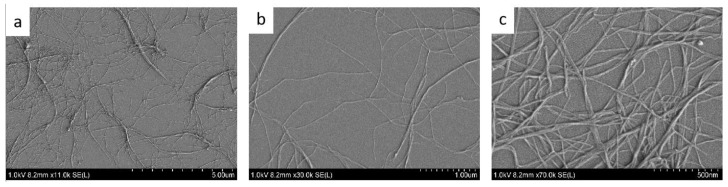
Scanning electron microscope (SEM) images of cellulose nanofibers (CNF) at different magnifications. (**a**) 11 k magnification, (**b**) 30 k magnification and (**c**) 70 k magnification of CNF.

SEM images of CNF (Figure 1) demonstrated the successful disintegration of tunicate cellulose by homogenization as indicated by elementary fibrils and a few microfibrillar aggregates. The CNF was rod-like in appearance with a width of 9.40 ± 1.54 nm and a length of 1.53 ± 0.41 to several µm, thus an aspect ratio greater than 163, consistent with previous studies [36]. Woody cellulose is the most common raw material to produce CNF. By using a similar technique, woody CNF was reported to possess an aspect ratio of ∼100 with dimensions of 2–60 nm in diameter and a few micrometers in length depending on the processing and pretreatment methods [37]. Due to its high aspect ratio, tunicate CNF is expected to be superior to woody CNF in terms of reinforcing effect [38]. The excellent reinforcing effect of tunicate CNF in composites has been demonstrated by adding tunicate CNF to epoxy resin-based composites, in which the addition of 16% tunicate CNF increased Young’s modulus from 1.6 GPa for the neat polymer film to 4.9 GPa for epoxy resin-tunicate CNF composite film [19]. 

**Figure 2:**
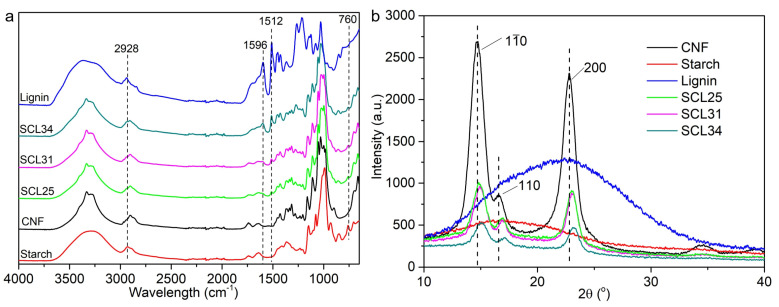
Fourier transform infrared (FTIR) (**a**) and X-ray diffraction (XRD) (**b**) spectra of starch, CNF, lignin and composite films.

CNF was very pure as indicated by its FTIR spectrum (Figure 2a). The peaks at 3334 and 2900 cm^−1^ were attributed to –OH stretching and C–H symmetrical stretching, respectively. The peaks at 1161 cm^−1^ and 1110 cm^−1^ originated from C–O anti-symmetric bridge stretching and C–OH skeletal vibration, respectively. The peaks at 1054 and 1031 cm^−1^ arose from the C–O–C pyranose ring skeletal vibration and the peak at 900 cm^−1^ generated from the glycosidic –CH deformation with a ring vibration and –OH bending in β-glycosidic linkages between glucoses [39]. These peaks are characteristic of pure cellulose [14,40]. The X-ray diffraction (XRD) patterns of CNF confirmed its cellulose I structure as indicated by characteristic peaks at 2θ 14.7°, 2θ 16.8° and 2θ 22.8° generating from plane (1ī0), plane (110) and plane (200), respectively (Figure 2b) [41]. CNF had a crystallinity index (CI) of 91.29%, agreeing well with the reported CI of 82–91% for tunicate CNF in literature [16]. 

### 3.2. Neat Film and Composite Film Preparation

Since starch, CNF and lignin could not be melted, the common methods used for thermoplastic polymers, such as extrusion or injection molding were not feasible for composite film preparation. Although Wu et al. (2009) [34] achieved films by dissolving cellulose, starch and lignin in ionic liquid, the costly solvent and complicated recycling procedure for ionic liquids will likely prohibit wide applications and implementation at commercial scale. In addition, the complete destruction of cellulose crystalline structure by dissolution resulted in deterioration of film performance, especially the mechanical and thermal properties. In this study, in order to avoid these negative effects, a low-cost and easily performed blending–casting–evaporation method was applied. When the solution/suspension was cast in petri dishes, and dried at 50 °C overnight flat films were formed by solvent evaporation.

**Figure 3:**
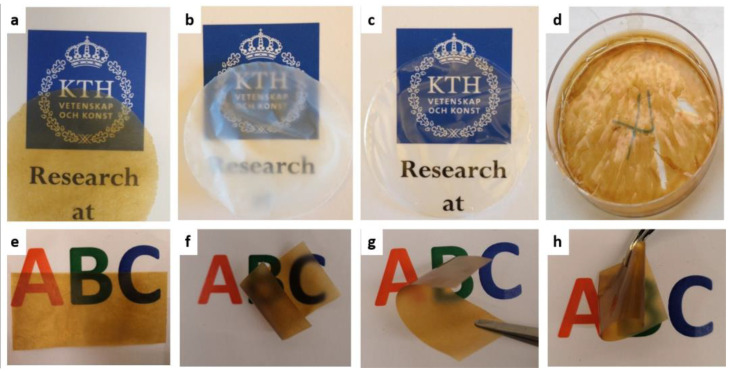
Photos of neat films and composite films made of starch, CNF and lignin. (**a**) SCL25 composite film, (**b**) CNF film, (**c**) starch film, and (**d**) no film formation for SL20, SL30 and SL40; (**e**–**h**), the flexibility and foldability of the SCL25 composite film.

Before casting, suspensions/solutions were prepared. Starch was dissolved in hot water and stirred overnight to generate a homogenous solution (0.5%). Acetone/water (4:1) was utilized to dissolve lignin at a designated concentration of 0.5%. Indeed, many other solvent systems had been recently used in lignin dissolution, such as harsh alkaline or acidic conditions and/or organic solvents [42,43,44]. However, these solvents were far from favorable and generally not considered environmentally friendly. Although ionic liquid was considered to be acceptable as a solvent for lignin, the major obstacle for its practical application is the high cost [42]. The acetone/water system has low environmental impact and high cost-efficiency, and it was also miscible with the water which is the solvent utilized to disperse or dissolve CNF and starch. Therefore, it was chosen as the best solvent for lignin in this work. By varying the ratios of starch, CNF and lignin (Table 1), a series of neat and composite films were prepared by using an identical blending-casting-evaporation procedure (Figure 3).

Lignin is considered as a rigid and brittle polymer with poor film-forming ability [45]. Therefore, when only lignin solutions were cast on the petri dishes, no film was obtained after solvent evaporation (data not shown). It has also been found that no film was formed if starch and lignin were blended, irrespective of mixing ratio, as indicated by cracks in the cast films (Figure 3d), due to the inherent brittleness and rigidity of lignin. Starch–lignin composites have been prepared either by casting or thermal molding [31], but noticeably different solvents were used for lignin, such as dimethyl sulfoxide and alkaline aqueous medium for water-soluble lignin and alkali lignin, respectively, rather than the acetone/water used in this study. However, when CNF was further introduced to the starch–lignin mixture, composite films were successfully prepared (Figure 3a). It is hypothesized that CNF maintains the integration of the films due to its similar hydrophilicity to starch and the natural fibrillar structure with high aspect ratio.

### 3.3. Neat Film and Composite Film Characterization

**Figure 4:**
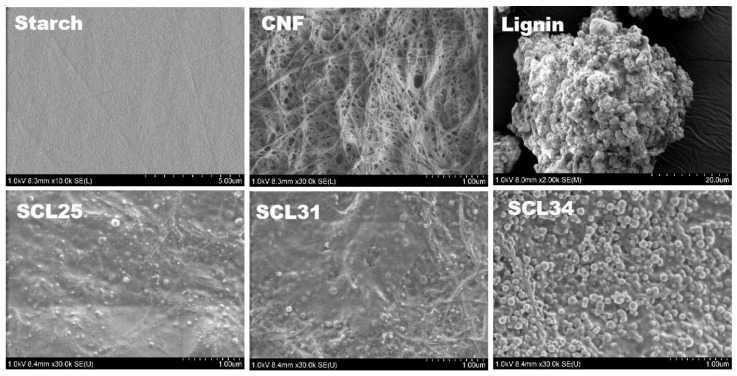
SEM images of neat films and composite films made of starch, CNF and lignin.

**Figure 5:**
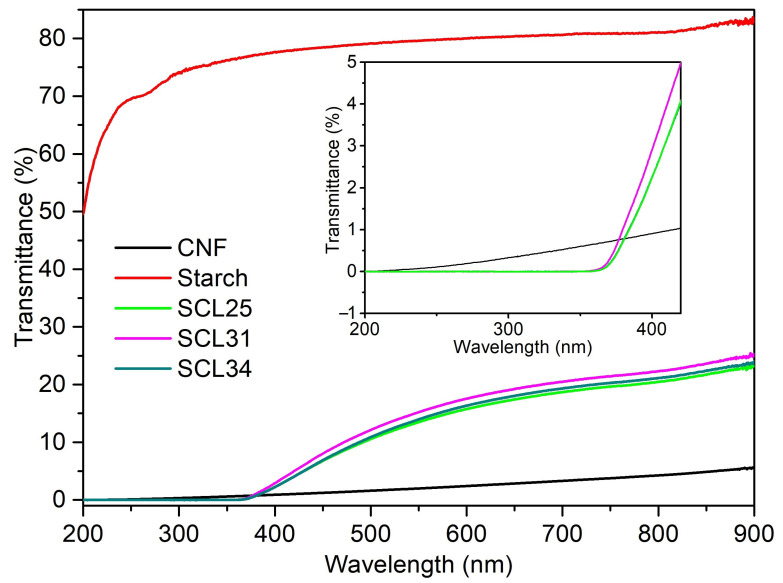
Transmittances of neat and composite films.

Neat starch films had a smooth surface (Figure 4) and were transparent (Figure 3c) with high transmittance of 80.20% at 750 nm (Figure 5). In general, starch films had poor mechanical properties as indicated by low tensile stress (32.95 MPa) and Young’s modulus (1.96 GPa) (Table 1). Due to the inherent hydrophilic characteristic of starch, the neat starch films showed a low contact angle of 18.78°. In contrast to starch films, neat CNF films were opaque with a lower transmittance of 3.66% (Figure 5). Generally, the formation of neat CNF films was considered as a self-assembly process, in which CNF fibrils randomly arranged to form mesh-like structures during drying. The thick and long fibrillar structure of CNF was preserved after film formation, resulting in numerous porous structures at the nanometer scale existing among the randomly packed CNF fibrils (Figure 4). CNF fibrils were flexible and had non-uniform distributions resulting in film surfaces that were uneven (Figure 4). Due to an extremely high Young’s modulus of 145–150 GPa for tunicate single cellulose fibrils [46] and the high aspect ratio of 163 for CNF in this study, neat CNF films showed good mechanical properties, as indicated by a tensile stress of 121.80 MPa, a Young’s modulus of 6.35 GPa and a tensile strain of 6.62% (Table 1). Similar to starch films, CNF films were also hydrophilic with a low contact angle of 24.20°.

Since CNF showed good tensile strength, it was expected that the addition of CNF into starch films could improve their mechanical performance. However, both starch and CNF are hydrophilic, which is not favorable for packaging applications. In order to overcome this drawback, another hydrophobic bio-polymer, lignin, was introduced to the starch–CNF films. To test this concept, a series of starch–CNF–lignin composite films with different formulations were prepared (Table 1). As expected, the addition of CNF improved the mechanical properties of the films. When starch and CNF were mixed 1:1, the obtained SC films had significantly improved mechanical properties compared with starch neat films: tensile stress of 140.23 MPa and Young’s modulus of 6.83 GPa, which were even superior to pure CNF films. This improvement could be due to the newly built-up hydrogen bonding between starch and CNF. On the other hand, CNF addition impaired the transmittance of starch films (11.21%) and the starch–CNF films remained hydrophilic with a contact angle of 57.20°. 

In order to improve the hydrophobicity of the composite, lignin ranging from 25% to 34% was introduced into the starch–CNF films. SCL25 films were brownish because of the presence of lignin (Figure 3a). SEM images confirmed that numerous lignin nanoparticles with the diameter of 50–250 nm were formed in the composite films (Figure 4). The formation of lignin nanoparticles probably results from the immiscibility of hydrophilic starch–CNF and hydrophobic lignin. Although lignin could be dissolved in acetone/water, when it was mixed with CNF in water, the ratio between acetone and water changed and was no longer suitable for lignin dissolution. This caused the lignin to aggregate in small droplets to form many nanoparticles after drying. Similar lignin nanoparticle formation was observed when lignin was incorporated into starch-based films [31]. By comparing the SEM images of SCL25, SCL31 and SCL34 in Figure 4, it was found that the size and density of lignin nanoparticle increased with increasing lignin percentage. Other than the lignin nanoparticles, the CNF’s fibrillar structure could still be clearly seen. 

The introduction of lignin into starch–CNF films was detected in FTIR spectra of the composite films (Figure 2a). Lignin showed many characteristic peaks including 1596 cm^−1^ arising from C=C stretching of aromatic ring, 1426 cm^−1^, 1452 cm^−1^ and 1512 cm^−1^ from aromatic ring vibration, 1264 cm^−1^ originating from aromatic ring breathing of the G unit and 1126 cm^−1^ resulting from the aromatic in-plane bending in the S unit [47]. When increasing lignin percentage from 25% to 34%, the densities of these characteristic peaks increased as well, confirming incorporation of lignin into these composite films. The interactions between these three components in the composite films were investigated by XRD and FTIR. In contrast to the crystalline structure of the CNF, starch was amorphous and no diffraction peak was observed in its XRD diffraction pattern (Figure 2b). A very broad peak around 22.5° was noted for lignin, indicating the semi-crystalline nature of lignin [48]. Although all the composite films showed similar diffraction patterns to the CNF, the addition of starch and lignin generated right-shifts of some characteristic peaks for CNF (Figure 2b), indicating the hydrogen bonds newly built up between these components. In addition, adding both starch and lignin lowered the densities of these peaks, which might arise from the shadowing effect of amorphous starch and semi-crystalline lignin.

**Figure 6:**
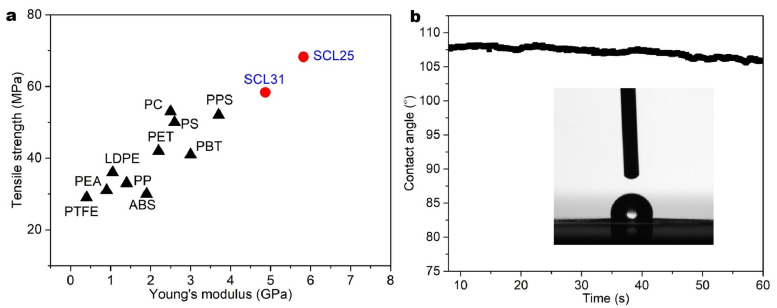
Mechanical properties (**a**) of different types of composite films and contact angles of SCL25 (**b**).

Lignin addition significantly reduced the mechanical strength of the films, as indicated by decreased tensile stress and Young’s modulus (Table 1). One contribution could be the incompatibility between the highly hydrophilic CNF and the hydrophobic lignin fraction, which generated some phase separation as indicated by lignin nanoparticle formation and lower mechanical strength. The hydrophobicity of the films was improved as indicated by the increased contact angle from 57° for SC films up to 107.50° for SCL25 composite films (Figure 6b) due to the presence of hydrophobic lignin nanoparticles. However, lignin is not the only factor determining the contact angle, since increasing lignin from 25% to 34% decreased the contact angle. As shown in the SEM evaluation of SCL34, abundant lignin nanoparticles made the film more porous, and this increased porosity facilitated water penetration, resulting in lower contact angles.

In UV regions (200–400 nm), starch only partially absorbed UV light and CNF did not completely block all UV light (Figure 5). However, the addition of lignin, irrespective of the amounts added, conferred essentially complete UV-blocking properties, with UV transmittance at nearly zero. This is due to the unique structure of lignin, derived from the polymerization of monolignols. In this process, electronic conjugation of the vinyl group para to the phenolic –OH is lost, generating UV chromophores at the coupling sites resulting in absorption in the UV range [49].

**Figure 7:**
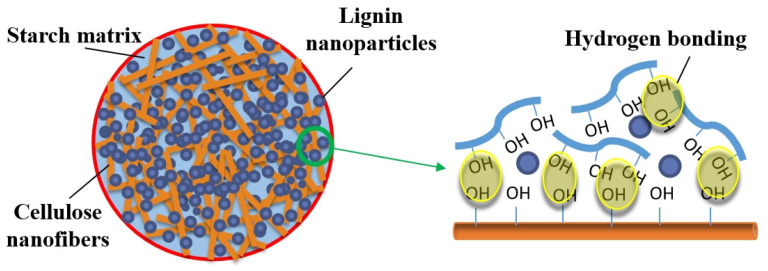
Schematic illustration of bonding mechanism of CNF, starch, and lignin in the biocomposites.

Based on the results presented above, the specific structures of the biocomposites could be illustrated as shown in Figure 7. In the composite films, the newly introduced hydrogen bonding between CNF and starch reinforced the structure, and lignin nanoparticles were entrapped in the network and gave the biocomposites good hydrophobicity. Due to the incorporation of lignin nanoparticles, the SCL films showed inferior mechanical properties to both neat CNF and SC films, although they were still significantly improved compared to neat starch films (Table 1). In addition, the tensile stress and the Young’s modulus of some composite films prepared in this study (SCL25 and SCL31), were comparable to or even higher than those of common plastics used for food packaging as shown in Figure 6a, supporting their acceptable mechanical properties for potential packaging applications. In addition, the composite films (e.g., SCL25), had very good flexibility and foldability (Figure 3e–h), which is also important in packaging applications.

### 3.4. Barrier and Thermal Properties of Composite Films

As found by O_2_ permeability tests, SCL25 films had oxygen permeability of 61.17 cm^3^·μm/m^2^·day·atm. Materials has previously been characterized as “high oxygen barrier” if its oxygen permeability is less than 75 cm^3^·μm/m^2^·day·atm at 25 °C and 50% of relative humidity [50]. Therefore, based on this definition, the SCL25 composite films is classified as a high oxygen barrier material. The high crystallinity of CNF (91%) could be one factor hindering the transport of oxygen through the material [51]. In addition, CNF fibrils will form organized structures and stack together closely due to hydrogen bonding. The addition of starch and lignin in this organized mesh structure may therefore yield low porosity which will resist passage of oxygen molecules. The low oxygen permeability of SCL25 suggests that it may be ideally suited for food packaging since this value is competitive with synthetic polymers such as PVC, which is one of the best barriers but contains chlorine atoms posing toxin risks at end-of-life incineration disposal [51].

**Figure 8:**
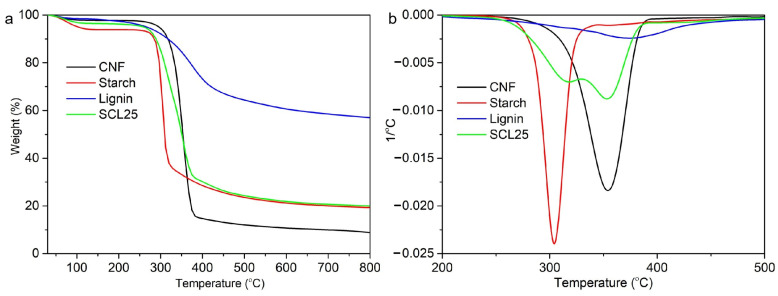
Thermo gravimetric (TG) (**a**) and derivative thermo gravimetric (DTG) (**b**) curves of starch, CNF, lignin and composite films.

Thermal stability and decomposition of neat films and composite films were determined using TGA in a nitrogen environment. TGA indicates weight loss and the first derivative (1st DTG) indicates the corresponding rate of weight loss (Figure 8). Both onset degradation temperature (T_o_) and peak degradation temperature (T_p_) can be presented as a measure of thermal decomposition and can be used as a means to compare the thermal stability characteristics of composite films. CNF films were the most thermally stable as indicated by the highest T_o_ and T_p_ values of 328.49 °C and 354.04 °C, respectively (Table 2). This was due to its highly crystalline structures. Starch films were more thermally stable than lignin, with T_o_ (292.66 °C) significantly higher than that for lignin (184.52 °C). However, lignin had a very high T_p_ of 377.05 °C, even greater than that for CNF (354.04 °C). Chemically, lignin is to a large extent composed of aromatic rings with various forms of branching. The complexity of chemical bonds in this structure leads to a wide range of degradation temperatures from 100 to 800 °C as indicated by its TGA curve [52]. Our TGA results for lignin were in agreement with these previous observations. After TGA measurement, 60% of lignin samples still remained un-volatized at 800 °C due to the formation of highly condensed aromatic structures which formed a char [53]. The SCL25 composite film showed a T_o_ of 294.77 °C. In contrast to the single degradation peaks observed for CNF, starch and lignin, the TGA curve of SCL25 was characterized by two degradation peaks (Figure 8b), T_p1_ at 326 °C and T_p2_ at 355 °C, which were close to the T_p_ of 305.77 °C and 354.04 for starch and CNF, respectively. Therefore, we propose that these two peaks were closely related to the degradation of starch and CNF components in the composite films. Compared to neat starch films, the thermal stability of the SCL25 composite film was improved as indicated by similar T_o_ and higher T_p_.

In summary, composite SCL25 films had a contact angle of 107.50°, an optical transmittance at 750 nm of 19.62%, UV blocking, a tensile stress of 68.26 MPa, a Young’s modulus of 5.82 GPa, an oxygen permeability of 61.17 cm^3^·μm/m^2^·day·atm and good thermal stability as indicated by high onset degradation temperature of 294.77 °C. To the best of our knowledge, only one publication has investigated the preparation of cellulose–starch–lignin composite films, which were made by dissolving these three components in ionic liquid followed by coagulation in water and drying [34]. In contrast to the lignin aggregation phenomenon observed in this study, the composite films after regeneration displayed uniformity from the interior to the surface, and no obvious phase separation could be seen. The prepared composite films after coagulation had tensile stresses of 14.5–35.2 MPa, significantly lower than 68.26 MPa for SCL25 in this study. During dissolution by ionic liquid, the crystalline I structure of cellulose is converted to cellulose II and the crystallinity index decreases, resulting in lower mechanical strength. Composite films prepared by ionic liquid dissolution had T_p_ of 331–335 °C, higher than T_p1_ of 312–320 °C for the composite films prepared in this study. This is probably due to the presence of cellulose II in the regenerated composite films, which was considered to be more thermally stable than cellulose I in the cast films obtained in this study. In addition, the oxygen permeability of our composite films was lower than the ~130 cm^3^·μm/m^2^·day·atm observed in the ionic liquid study, which might be due to the preserved crystalline structure and organized fiber of the CNF. It has been reported that crystalline cellulose could significantly decrease the oxygen permeability of PLA-based films, because it had the ability to form hydrogen bonds resulting in strong networking that made the composite films resistant to molecular passage. 

## 4. Conclusions

In this study, novel starch–CNF–lignin composite films were successfully prepared by a facial blending–casting–evaporation method. The high performance of the composite films was achieved by simply tuning the formulation of the three components without any chemical modification or reaction. CNF and starch showed good miscibility, and the lignin was distributed as nanoparticles on the surface or incorporated into the starch-cemented structure. The hydrogen bonds newly built up among these three components improved their integration in the composite films. CNF made a substantial contribution to the mechanical strength of the starch-based film. The introduction of hydrophobic lignin significantly improved the hydrophobicity of the composite films and provided UV absorbance. The prepared films also showed improved thermal stability and oxygen barrier properties due to the presence of highly crystalline CNF. With the optimal formulation found in this study, CNF–starch–lignin composite films are flexible, showing many desired properties including: transparency (optical transmittance at 750 nm of 19.62%), hydrophobicity (contact angle of 107.50°), complete UV-blocking, high mechanical strength (tensile stress of 68.26 MPa and Young’s modulus of 5.82 GPa), gas barrier (oxygen permeability of 61.17 cm^3^·μm/m^2^·day·atm) and thermal stability (onset degradation temperature of 294.77 °C). These properties establish such films as potentially suitable, sustainable alternatives to petroleum-based plastics in food packaging.

## Figures and Tables

**Table 1:** Formulation and characterization of neat and composite films made of starch, CNF and lignin.

	Formulation			Mechanical Properties			Hydrophobicity	Optical Properties
	Starch (%)	CNF (%)	Lignin (%)	Tensile Stress (MPa)	Young’s Modulus (GPa)	Tensile Strain (%)	Contact Angle (°)	Transmittance (%)
CNF	0	100	0	121.80	6.35	6.62	24.20	3.66
Starch	100	0	0	32.95	1.96	8.72	18.78	80.20
SC	50	50	0	140.23	4.90	6.83	57.20	11.21
SL20	80	0	20	No film formed.				
SL30	70	0	30	No film formed.				
SL40	60	0	40	Starch aggregation. No film formed.				
SCL25	42	33	25	68.26	5.82	2.12	107.50	19.62
SCL31	34.5	34.5	31	58.39	4.87	1.75	81.83	20.29
SCL34	33	33	34	22.71	4.74	0.46	56.16	17.08

**Table 2:** Thermal properties of neat films and composite films (°C).

**Neat Films**	T_o_ *	T_p_ *	
CNF	328.49	354.04	
Starch	292.66	305.77	
Lignin	184.52	377.05	
**Composite Films**	**T_o_**	T_p1_ *	T_p2_ *
SCL25	294.77	326.00	355.50

* T_o_, onset degradation temperature; T_p_, peak degradation temperature; T_p1_, first peak degradation temperature and T_p2_, second peak degradation temperature.
